# Using Multivariable Mendelian Randomization to Disentangle the Causal Effects of Lipid Fractions

**DOI:** 10.1371/journal.pone.0108891

**Published:** 2014-10-10

**Authors:** Stephen Burgess, Daniel F. Freitag, Hassan Khan, Donal N. Gorman, Simon G. Thompson

**Affiliations:** Cardiovascular Epidemiology Unit, Department of Public Health and Primary Care, University of Cambridge, Cambridge, Cambridgeshire, United Kingdom; University Medical Center Utrecht, The Netherlands

## Abstract

**Background:**

Previous Mendelian randomization studies have suggested that, while low-density lipoprotein cholesterol (LDL-c) and triglycerides are causally implicated in coronary artery disease (CAD) risk, high-density lipoprotein cholesterol (HDL-c) may not be, with causal effect estimates compatible with the null.

**Principal Findings:**

The causal effects of these three lipid fractions can be better identified using the extended methods of ‘multivariable Mendelian randomization’. We employ this approach using published data on 185 lipid-related genetic variants and their associations with lipid fractions in 188,578 participants, and with CAD risk in 22,233 cases and 64,762 controls. Our results suggest that HDL-c may be causally protective of CAD risk, independently of the effects of LDL-c and triglycerides. Estimated causal odds ratios per standard deviation increase, based on 162 variants not having pleiotropic associations with either blood pressure or body mass index, are 1.57 (95% credible interval 1.45 to 1.70) for LDL-c, 0.91 (0.83 to 0.99, p-value  = 0.028) for HDL-c, and 1.29 (1.16 to 1.43) for triglycerides.

**Significance:**

Some interventions on HDL-c concentrations may influence risk of CAD, but to a lesser extent than interventions on LDL-c. A causal interpretation of these estimates relies on the assumption that the genetic variants do not have pleiotropic associations with risk factors on other pathways to CAD. If they do, a weaker conclusion is that genetic predictors of LDL-c, HDL-c and triglycerides each have independent associations with CAD risk.

## Introduction

Mendelian randomization employs genetic variants to estimate the causal effect of a risk factor on a disease. It is based on the principle that the distribution of a particular genetic variant in a population is analogous to the allocation of treatment in a randomized controlled trial [1]. For valid causal conclusions, each genetic variant used in a Mendelian randomization analysis must be only associated with the risk factor of interest, and be independent of all confounding variables. There must also be no causal pathway leading from the genetic variant to the disease, except for that through the risk factor of interest. These assumptions define an instrumental variable [2]. An association between such a genetic variant and the disease implies that the risk factor has a causal effect on the disease, analogous to inferring an intention-to-treat effect from an association between randomization and disease in a randomized controlled trial [3]. If the assumptions are violated, then a non-zero Mendelian randomization estimate still provides evidence that the risk factor and disease share common genetic predictors, but the estimated causal effect will be biased.

If there are several related risk factors, instrumental variable methods can be used to estimate the causal effects of each of the risk factors in a single analysis model [4]. Multiple genetic variants are required which are analogous to multiple treatment assignments in a factorial randomized trial, and have different magnitudes of association with each of the risk factors. Even if none of the variants show specific associations with any individual risk factor, causal assessments can be made by comparing the associations of the variants with each of the risk factors simultaneously. This approach has been referred to as ‘multivariable Mendelian randomization’ [5].

**Figure 1:**
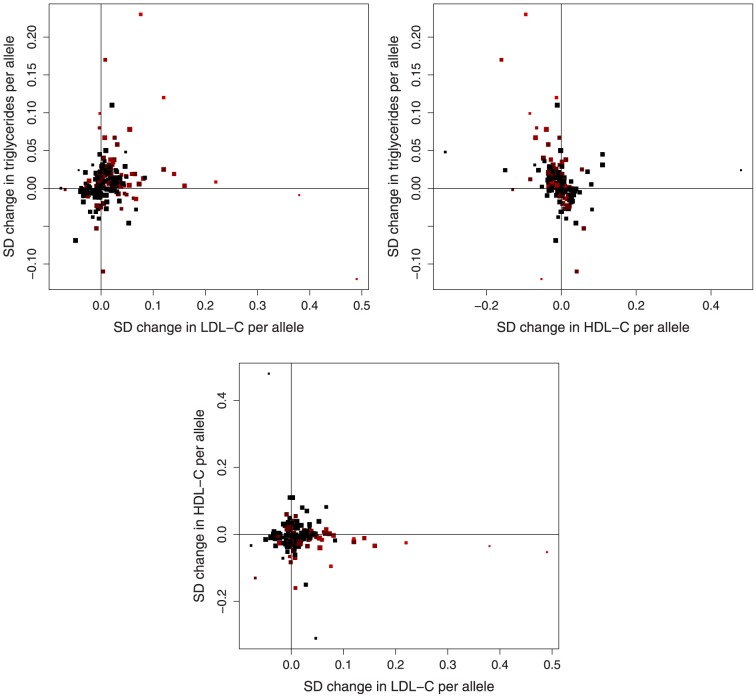
Associations of lipid-related genetic variants with lipid fractions. Association of coronary artery disease (CAD) risk-increasing alleles of 185 genetic variants with all pairs of low-density lipoprotein cholesterol (LDL-c), high-density lipoprotein cholesterol (HDL-c), and triglycerides (brightness and size of points: brighter points correspond to stronger associations with CAD risk, larger points correspond to more precise estimates). Note that some points are overlapping.

**Figure 2:**
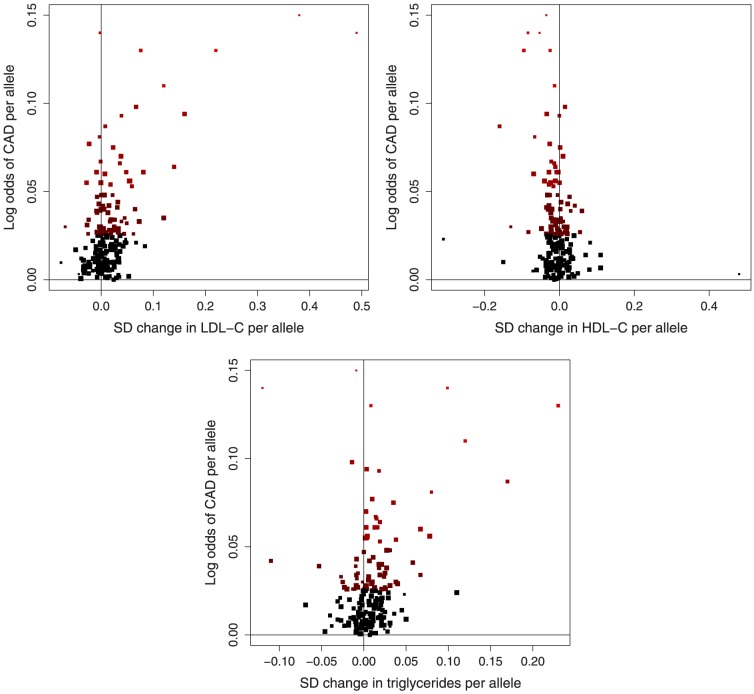
Associations of lipid-related genetic variants with lipid fraction and CAD risk. Association of coronary artery disease (CAD) risk-increasing alleles of 185 genetic variants with odds of CAD, and with each of low-density lipoprotein cholesterol (LDL-c), high-density lipoprotein cholesterol (HDL-c) and triglycerides in turn (brightness and size of points: brighter points correspond to stronger associations with CAD risk, larger points correspond to more precise estimates). Note that some points are overlapping.

We consider 185 variants having known associations with at least one of low-density lipoprotein cholesterol (LDL-c), high-density lipoprotein cholesterol (HDL-c) and triglycerides in 188,578 participants reported by the Global Lipids Genetics Consortium [6]. We combine these with data on the associations of these variants with the risk of coronary artery disease (CAD) based on 22,233 cases and 64,762 controls taken from the CARDIoGRAM consortium [7]. The multivariable Mendelian randomization approach was taken as there are few genetic variants associated with one lipid fraction which are not also associated with a further lipid fraction, particularly for triglycerides. The data are displayed graphically in Figures 1–2.

These data were previously investigated by Do et al., who performed a number of *ad hoc* analyses to try to disentangle the causal relationships of the lipid fractions [8]. These suggested that, while LDL-c and triglycerides are both causally related to CAD risk, HDL-c is not. However, their regression-based approaches do not account for statistical uncertainty in the reported genetic associations leading to potentially incorrect inference. They also allow information from each variant to receive an equal weight in the analysis, rather than the more common variants receiving a greater weight. This leads to potential bias and inefficiency in the causal estimates, as demonstrated in a simulation study [5]. Additionally, correlation between the genetic variants was not accounted for in the analysis. The conclusions of Do et al. are therefore in doubt.

A multivariable Mendelian randomization analysis requires that the genetic variants used are:

1. associated with the risk factors of interest (here LDL-c, HDL-c and triglycerides),
2. not associated with confounders of any of the associations of the risk factors of interest with the disease (here CAD),
3. not associated with the disease except via pathways through the risk factors of interest.

**Figure 3:**
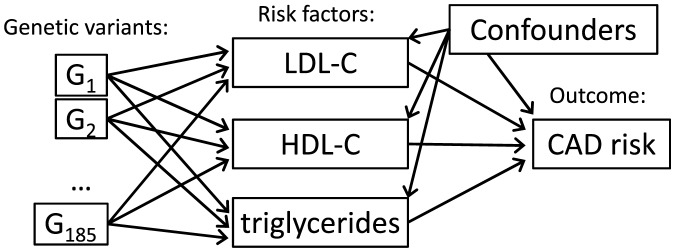
Causal assumptions as a directed acyclic graph. Diagram of causal relationships between genetic variants, risk factors (low-density lipoprotein cholesterol, LDL-c; high-density lipoprotein cholesterol, HDL-c; and triglycerides), confounders, and disease (coronary artery disease, CAD). Although confounders (common causes of a risk factor and the outcome) are represented as a single variable for simplicity, each risk factor may have a different set of confounders.

These assumptions (Figure 3), which are also implicit in the methods of Do et al., correspond with those of an instrumental variable in a conventional Mendelian randomization analysis. It is not necessary for each genetic variant to be associated with all of the risk factors of interest, but estimates of the causal effects of the risk factors will be imprecise unless there are a number of variants which have differing magnitudes of association with each of the risk factors.

We use a likelihood-based statistical method for analyzing summarized data on the genetic associations with the risk factors and with disease [9] extended for application to multiple risk factors. The likelihood-based method was performed in a Bayesian analysis framework using WinBUGS v1.4.3 [10]. Further details of the analysis are provided in the Methods and Models section.

## Results

The results of our likelihood-based method are contrasted with those from Do et al. in Table 1 (first two rows). Our results for LDL-c and triglycerides are qualitatively similar to those of Do et al., although the magnitude of the causal odds ratio for LDL-c is slightly greater and that for triglycerides slightly less. For HDL-c, however, we find a statistically significant inverse causal effect (P = 0.008) which Do et al. did not. Its magnitude is less than those for LDL-c and triglycerides, which may be why our more statistically rigorous analysis (which allows appropriate weighting of variants based on the precision of their associations with the risk factors and outcome) was able to identify it.

**Table 1:** Causal odds ratios (95% confidence/credible intervals) of coronary artery disease per standard deviation increase in each lipid fraction (low-density lipoprotein cholesterol, LDL-c; high-density lipoprotein cholesterol, HDL-c; and triglycerides), with two-sided p-value for HDL-c.

Method	Number of variants	LDL-c	HDL-c	Triglycerides	p-value for HDL-c
Do et al. a	185	1.46 (1.37 to 1.57)	0.96 (0.89 to 1.03)	1.43 (1.28 to 1.61)	0.35
Multivariable MR	185	1.53 (1.42 to 1.66)	0.90 (0.82 to 0.97)	1.33 (1.20 to 1.47)	0.008
Multivariable MR	162 b	1.57 (1.45 to 1.70)	0.91 (0.83 to 0.99)	1.29 (1.16 to 1.43)	0.027

MR  =  Mendelian randomization.

a Derived from Table 3 of Do et al. [8]

b Removing 23 variants having known pleiotropic associations with blood pressure or body mass index.

The crucial assumption necessary for a causal interpretation of our estimates is that the genetic variants used do not have pleiotropic effects with confounders on other pathways to CAD. It is never possible to fully investigate this assumption, but we have matched the 185 genetic variants used against the GWAS catalogue [11] and supplementary material provided by the Global Lipids Genetics Consortium [6] and identified 23 of them which have an association (P<0.05) with systolic or diastolic blood pressure, or with body mass index. Removing these 23 genetic variants, and repeating the multivariable Mendelian randomization analysis (Table 1, third row), gave similar estimates to our previous analysis, although the resulting p-value of 0.027 for HDL-c might not be regarded as definitively conclusive.

**Figure 4:**
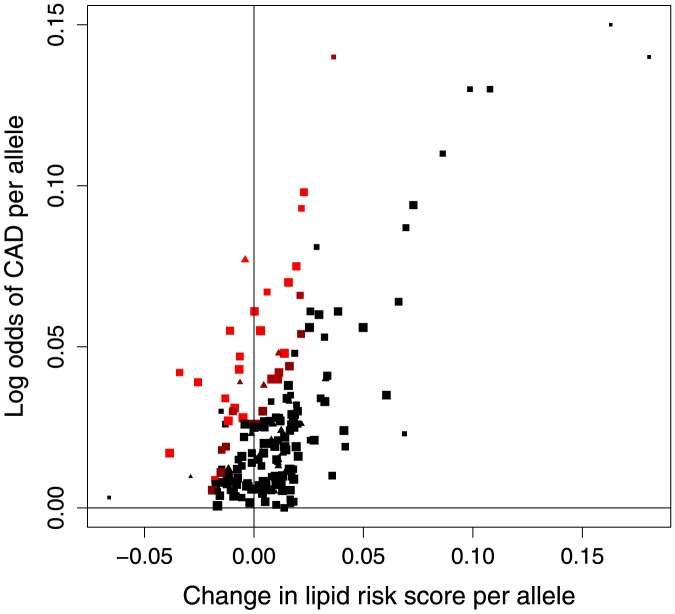
Association of lipid score for all lipid-related genetic variants with CAD risk. Association of coronary artery disease (CAD) risk-increasing alleles of 185 genetic variants with lipid risk score and odds of CAD (brightness corresponds to percentile of chi-squared distribution for heterogeneity test: 98th or higher [brightest red], 95th to 98th, 90th to 95th, below 90th [black]). Note that some points are overlapping. Variants associated with blood pressure or body mass index (P<0.05) are displayed as triangles.

A heterogeneity test indicated that there was more variability in the genetic associations with the outcome than could be explained by chance alone (185 variants, P = 2×10^−18^, 162 variants P = 1×10^−16^). This test is analogous to an over-identification test usually performed in a conventional instrumental variable analysis with individual-level data [12]. Such heterogeneity may partly be explained by misspecification of the model relating the risk factors and the outcome (for example, departure from linearity), but it is likely that there is residual pleiotropy in many of the genetic variants. Heterogeneity can also be assessed visually by plotting the observed and expected associations with CAD risk for each genetic variant using a lipid score based on the associations of the variant with the lipid fractions (Figure 4). The lipid score for a given variant is a linear function of the genetic associations with each of the lipid fractions multiplied by the relevant causal effect estimate. This reflects the expected association of each variant with CAD risk based on the causal effects of the lipid fractions. If the genetic variants are only associated with the outcome via the risk factors under analysis, this graph should (apart from random variation) be a straight-line through the origin; deviation from this is an indication of pleiotropy.

*Post hoc* analyses were also performed where genetic variants were removed from the analysis if their contribution to the heterogeneity test was greater than the 98th, 95th and 90th percentiles of the relevant chi-squared distribution. Pruning variants at the 98th percentile, 24 variants were excluded from the analysis; at the 95th percentile, 7 more variants were excluded; at the 90th percentile, 11 further variants were excluded. The heterogeneity test statistic was greatly reduced (P = 0.16, 0.62, 0.97 at 98th, 95th, 90th percentile). Similar results for the causal effect estimate of HDL-c were observed pruning at the 98th percentile (odds ratio 0.91, 95% credible interval 0.84 to 0.99, P = 0.030), although the estimate for HDL-c attenuated towards the null when pruning variants at the 95th (0.93, 0.85 to 1.01, P = 0.086) and 90th percentiles (0.94, 0.86 to 1.02, P = 0.13). The *post hoc* results show some robustness of the finding that HDL-c-associated variants predict CAD risk, although the strength of association reduces as more variants are excluded from the analysis.

## Discussion

Mendelian randomization analyses that use a single genetic variant, for which the biological function is understood, are easily interpreted, as it is clear how the variant relates to the change in the risk factor. They are also valuable in that the function of the variant often provides a clue as to clinical or pharmaceutical interventions that may have a corresponding effect on the disease. When multiple variants for the same risk factor are used, each variant will have a specific functional pathway by which it is associated with the risk factor. The overall causal estimate reflects a weighted average change in the disease risk resulting from long-term interventions in the risk factor. However, it may be that not all ways of intervening on the risk factor result in the same magnitude of change in the disease. Therefore, it would be misleading to assume that all interventions on LDL-c, HDL-c and triglycerides would result in changes in the risk of CAD. As the functions of all the variants and the causal pathways between the risk factors (both measured and unmeasured) are unknown, we prefer to state that, for example, triglyceride-related pathways have a causal role in CAD risk, rather than necessarily triglycerides themselves.

If the extended instrumental variable assumptions necessary for multivariable Mendelian randomization are not satisfied for one or more of the variants, then the causal effect estimates may be biased. Systematic bias would not be expected if pleiotropic associations of the variants (beyond those with LDL-c, HDL-c and triglycerides) are balanced between those which are beneficial and harmful for CAD risk [13]. However, even if the assumptions are violated and there is bias, this analysis still indicates a shared genetic architecture for CAD risk comprising independent components associated with each of LDL-c, HDL-c and triglycerides.

The interpretation of the causal estimates derived for each risk factor of interest on the disease also depends on whether or not the risk factors are themselves causally dependent. For example, if the effect of one risk factor on the disease is wholly or partially mediated by another risk factor, then they are causally dependent. In Figure 3, the lack of arrows between the risk factors indicates the assumption that these risk factors are causally independent. In fact, there is some biological and epidemiological evidence that the effect of triglycerides on CAD risk is mediated by HDL-c and LDL-c levels [14], [15]. If there are causal effects between the risk factors, then estimates from a multivariable Mendelian randomization approach will represent the direct causal effects of each of the risk factors on the disease, not including indirect pathways via the other risk factors [5]. These will typically differ from the total effect of the risk factors. However, they still provide evidence on the causal involvement of the risk factors even if another part of the causal effect is mediated via another risk factor.

Additionally, the consideration of only three lipid categories is a simplification [16]. Some lipid fractions (for example, intermediate-density lipoprotein cholesterol) are omitted in the analysis, and the variability of particle size within the categories is ignored. If the associations with further lipid categories or a finer categorization of lipid fractions were measured, then these could be included in an analysis. However, the identification of the causal effects of precisely defined risk factors is only possible if the function of the genetic variants in the analysis is known. In this case, with multiple genetic variants having (in some cases) unknown functions, it is more appropriate to concentrate on whether the risk factor has a causal interpretation and the direction of the causal effect. For this reason, the division into a small number of clinically-relevant lipid categories is preferred.

Previous Mendelian randomization analyses for HDL-c have not reported statistically significant findings [17], although this may reflect a lack of statistical power due to the low proportion of variation in HDL-c explained by the limited number of genetic variants used. For example, the *LIPG* variant examined by Voight *et al*. was associated with a 0.29 standard deviation change in HDL-c and had a population frequency of 2.6%, so that the variant explained 0.2% of the variance of HDL-c (*R^2^* = 0.002). This translates to less than 10% power to detect an odds ratio of 0.90 per standard deviation increase in HDL-c even in the sample size of 20,913 cases and 95,407 controls considered by the authors [18]. Their analysis using 14 variants associated with HDL-c but not with LDL-c or triglycerides (P>0.05) gave an odds ratio estimate of 0.93 per standard deviation increase in HDL-c, although with a wide confidence interval (95% confidence interval 0.68, 1.26). Another recent study found that a genetic score for HDL-c calculated using all genetic variants associated with HDL-c at a GWAS level of significance was associated with CAD risk, while a similar score for HDL-c excluding those variants associated with LDL-c or triglycerides at a nominal level of significance (P>0.05) was not associated with CAD risk [19]. However, this restricted score explained only a small proportion of the variance in HDL-c (0.3%), and was also associated with LDL-c and triglycerides, limiting the utility of this analysis. Although the authors showed no association between the unrestricted genetic score for HDL-c and CAD risk on adjustment for LDL-c and triglycerides, these variables are on alternative causal pathways from the genetic variants to the outcome, meaning that such adjustment is inappropriate and may lead to bias [20].

Pharmaceutical agents which raise HDL-c levels have failed to show benefits for CAD and total mortality outcomes in clinical trials [21], [22]. As our analysis uses many genetic variants with different functions, it is not specifically informative about the efficacy of an intervention for HDL-c on a particular causal pathway. Additionally, causal effect estimates from Mendelian randomization tend to overestimate the proportional effect of clinical interventions [23]. For example, statin therapy in the primary prevention of CAD over five years reduces LDL-c by around 30% and CAD risk by 27% (95% confidence interval 23, 30%) [24]. In contrast, genetic variants specifically associated with LDL-c predict a 67% (54, 76%) reduction in CAD risk for a 30% reduction in LDL-c [9]. Hence, even if HDL-c related pathways are causal for CAD, the expected magnitude of effect from clinical intervention may be much lower than the 9% decrease in CAD risk per 1 standard deviation predicted by the genetic analysis.

Multivariable Mendelian randomization is likely to be a promising design strategy for investigating the causal effects of closely-related risk factors with common genetic predictors. It enables a Mendelian randomization analysis of a risk factor even if there are no variants solely associated with it, such as triglycerides in this example. However, inference of a causal effect relies on the differential associations of multiple genetic variants with the disease, and so cannot be obtained from the distribution of a single genetic variant. Consequently, the intuitive appeal of using Mendelian randomization to infer a causal effect from a variant's sole associations with the risk factor and disease is somewhat reduced.

In conclusion, our analyses support the conclusions of Do et al. that LDL-c and triglycerides are independent risk factors for CAD, but additionally suggest that HDL-c-related pathways may also have a causal role in CAD.

## Methods and Models

Estimates for the causal effects of LDL-c, HDL-c and triglycerides on the risk of CAD are obtained using data on the beta-coefficients and standard errors from the regression of the variables on each of the genetic variants in turn. These coefficients were taken from Do et al. [8], who reported the beta-coefficients from linear regression for the lipid fractions (scaled per 1 standard deviation increase in the lipid fraction) and from logistic regression for the disease outcome. Standard errors were obtained from the p-values cited in the paper; if the p-value was exactly 1 (which occurred 2 times out of 740 associations when the beta-coefficient was 0), the average of the standard errors for that variable across the other variants was taken. The standard errors were obtained using R [25], except when the p-value was lower than the smallest non-zero normalized floating-point number allowed in R (around 2×10^−308^), in which case Wolfram Alpha was used (this occurred for 2 p-values) [26].

In the likelihood-based method, a multivariate normal distribution is assumed for the beta-coefficients representing the genetic associations with each of the risk factors and the disease. We assume that the estimate of association of genetic variant *j*, *j* = 1, 2, …, 185 with LDL-c is *X_Lj_* with standard error *σ_Lj_*, and similarly with HDL-c (*X_Hj_*, standard error *σ_Hj_*), triglycerides (*X_Tj_*, standard error *σ_Tj_*) and with odds of CAD (*Y_j_*, standard error *σ_Yj_*):
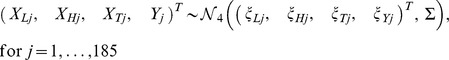


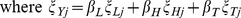


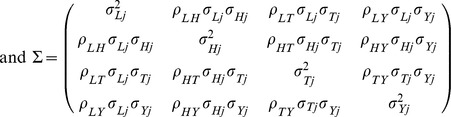



The parameters *β_L_*, *β_H_*, and *β_T_* are the causal effects of LDL-c, HDL-c, and triglycerides on CAD risk. As the beta-coefficients for the disease (*Y_j_*) are log odds ratios, the causal parameters represent log odds ratios for a unit increase (here, scaled to be a 1 standard deviation increase) in the risk factor. If the genetic variants were independent, then the overall likelihood would be the product of the above likelihood contributions for each genetic variant. As some of the genetic variants are correlated in their distributions (that is, in linkage disequilibrium), correlations are allowed in the likelihood contributions for these variants by assuming a multivariate normal distribution for all the coefficients of the correlated variants. The elements in the variance-covariance matrix are obtained using the correlations between genetic variants (as these correlations should be equal to the correlations between the beta-coefficients for the same variables). These correlations were taken from the 1000 Genomes Pilot 1 dataset and obtained from the SNP Annotation and Proxy Search (SNAP; http://www.broadinstitute.org/mpg/snap/ldsearchpw.php), and were all less than 0.25.

Causal estimates can be evaluated by numerical maximization of this likelihood function, or by Bayesian methods. Here, direct maximization of the likelihood is impractical, as there are 558 ( = 185 × 3 + 3) parameters to optimize over. The analysis was therefore performed in a Bayesian framework using WinBUGS. Vague normal priors with mean zero and variance 10002 were used for each of the unknown parameters. Uniform priors for the *β_L_*, *β_H_*, and *β_T_* parameters on [−3, +3] and on [−2, +2] were also considered; identical results were obtained. The use of vague priors corresponds to no external evidence being incorporated in the analysis, and means that the posterior distribution of the parameters approximates the frequentist likelihood function. The Bayesian method is undertaken purely for computational reasons, and does not correspond to a subjective Bayesian analysis.

Rather than maximizing the likelihood function, we take a sample from the posterior distribution using Monte Carlo Markov Chain (MCMC) sampling. We regard the mean and standard deviation of the posterior distribution as the ‘estimate’ and ‘standard error (SE)’. Twice the tail probability from the posterior distribution for the parameter having the opposite sign to the point estimate is regarded as the two-sided ‘p-value’. Further details of the method, including a simulation study, have been previously published [5].

In the likelihood function, the correlation between the beta-coefficients for LDL-c and HDL-c (*ρ_LH_*) was taken as −0.1, for LDL-c and triglycerides (*ρ_LT_*) 0.2, for LDL-c and CAD risk (*ρ_LY_*) 0.1, for HDL-c and triglycerides (*ρ_HT_*) −0.1, for HDL-c and CAD risk (*ρ_HY_*) −0.1, and for triglycerides and CAD risk (*ρ_TY_*) 0.1. These values were taken as estimates of the correlations between the variables LDL-c, HDL-c, triglycerides, and the log odds of disease risk, which will be similar to the correlations between the beta-coefficients used in the likelihood-based analysis. A sensitivity analysis for the values of the correlation parameters (*ρ..*) is given in Table 2. As well as the original parameter values (shown in italics), we take parameter values 2, 1.5, 0.5, 0, −0.5 and −1 times these values. We see that the causal estimates and standard errors are robust to different choices of these parameters.

**Table 2:** Sensitivity analysis for the correlation parameters (*ρ*_.._) between beta-coefficients for genetic associations with LDL-c (*β_L_*), HDL-c (*β_H_*), triglycerides (*β_T_*) and CAD risk (*β_Y_*): estimates of causal log odds ratios *β_L_*, *β_H_*, and *β_T_* (with standard errors).

*ρ_LH_*	*ρ_LT_*	*ρ_LY_*	*ρ_HT_*	*ρ_HY_*	*ρ_TY_*	*β_L_*	*β_H_*	*β_T_*
−0.2	0.4	0.2	−0.2	−0.2	0.2	0.42 (0.04)	−0.10 (0.04)	0.28 (0.04)
−0.15	0.3	0.15	−0.15	−0.15	0.15	0.42 (0.04)	−0.11 (0.04)	0.28 (0.04)
−0.1	0.2	0.1	−0.1	−0.1	0.1	0.43 (0.04)	−0.11 (0.04)	0.28 (0.04)
−0.05	0.1	0.05	−0.05	−0.05	0.05	0.43 (0.04)	−0.11 (0.04)	0.28 (0.04)
0	0	0	0	0	0	0.43 (0.04)	−0.12 (0.04)	0.29 (0.04)
0.05	−0.1	−0.05	0.05	0.05	−0.05	0.43 (0.04)	−0.12 (0.04)	0.29 (0.04)
0.1	−0.2	−0.1	0.1	0.1	−0.1	0.44 (0.04)	−0.12 (0.04)	0.29 (0.04)

To assess the homogeneity of the causal effects of the risk factors assessed using different genetic variants, we performed a likelihood-ratio test of the hypothesis that the causal effects of the risk factors were the same for each genetic variant (H_0_: *ξ_Yj_* = *β_L_ ξ_Lj_*+*β_H_ ξ_Hj_*+*β_T_ ξ_Tj_* for all *j*), versus the alternative hypothesis that the genetic associations with the outcome for each of the genetic variants were unrestricted (H_1_: *ξ_Yj_* unrestricted). The likelihood functions were calculated at the relevant point estimates (the mean of the posterior distribution); twice the difference in the log-likelihood function was then compared to a chi-squared distribution with the appropriate number of degrees of freedom. For the *post hoc* analysis, the likelihood contributions for each linkage disequilibrium-block of variants were considered separately and compared to the 98th, 95th, and 90th percentiles of the relevant chi-squared distribution. With 185 variants, the test statistic was 403.4 (P = 8×10^−19^ for a chi-squared distribution with 185–3 = 182 degrees of freedom); with 162 variants (excluding variants associated with blood pressure or body mass index), the test statistic was 352.2 (P = 1×10^−16^; 159 degrees of freedom).

In the *post hoc* analyses excluding variants having heterogeneous association with the outcome, the test statistics were 175.2 (P = 0.17; 158 degrees of freedom) pruning at the 98th percentile; 145.1 (P = 0.62; 151 degrees of freedom) pruning at the 95th percentile; and 111.1 (P = 0.97; 140 degrees of freedom) pruning at the 90th percentile.

The genetic variants omitted from the analysis due to possibly pleiotropic associations with either blood pressure or body mass index are: rs4660293, rs2710642, rs2290547, rs3822072, rs13107325, rs6450176, rs1800562, rs998584, rs702485, rs2293889, rs1883025, rs2068888, rs12801636, rs653178, rs4983559, rs2652834, rs3198697, rs2000999, rs2925979, rs731839, rs492602, rs181362, and rs5763662. These variants are displayed in Figure 4 as triangles.

The genetic variants omitted from the analysis on a *post hoc* basis due to heterogeneity are: rs10493326, rs4587594, rs2642438, rs903319, rs7422339, rs1250229, rs1515110, rs7640978, rs2240327, rs2602836, rs4976033, rs205262, rs12525163, rs17145738, rs799160, rs4921914, rs8176720, rs579459, rs653178, rs6489818, rs1186380, rs1169288, rs4465830, and rs3761445 in the analysis pruning at the 98th percentile; additionally rs17345563, rs998584, rs3996352, rs2412710, rs9930333, rs8077889, and rs4148005 in the analysis pruning at the 95th percentile; additionally rs2290547, rs9686661, rs4917014, rs2326077, rs4871137, rs7832643, rs1781930, rs10832962, rs12801636, rs8017377, and rs952044 in the analysis pruning at the 90th percentile. These variants are displayed in Figure 4 using different colours: the brightest points are those pruned at the 98th percentile.

### Data visualization

To aid with visualizing the data, we provide several plots of the genetic associations with the lipid fractions and with CAD risk. Each of the genetic variants is orientated such that the “risk-increasing allele” (the allele associated with an increased risk of CAD) is displayed in each case. Figure 1 shows the per allele associations with each of the lipid fractions in pairs. Figure 2 shows the per allele associations with CAD risk and each of the lipid fractions. Figure 4 shows the association of a lipid score, calculated by multiplying the per allele associations with each of the lipid fractions by the causal effect estimate of the lipid fraction on CAD risk. The lipid score for variant j is therefore:
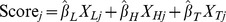



If there is no pleiotropy and the causal effects corresponding to the different variants of each of the lipid fractions are homogeneous, then (apart from random variation) this graph should be a straight line through the origin.

Interactive versions of these graphs can be found and explored at http://www.phpc.cam.ac.uk/charttest3.html.
